# Oral and Vaginal Hormonal Contraceptives Induce Similar Unfavorable Metabolic Effects in Women with PCOS: A Randomized Controlled Trial

**DOI:** 10.3390/jcm12082827

**Published:** 2023-04-12

**Authors:** Maria-Elina Mosorin, Terhi Piltonen, Anni S. Rantala, Marika Kangasniemi, Elisa Korhonen, Risto Bloigu, Juha S. Tapanainen, Laure Morin-Papunen

**Affiliations:** 1 Department of Obstetrics and Gynecology, Oulu University Hospital, Wellbeing Services County of North Ostrobothnia, 90220 Oulu, Finland; 2 Research Unit of Clinical Medicine, University of Oulu, 90220 Oulu, Finland; 3 Medical Research Center, University of Oulu, Oulu University Hospital, Wellbeing Services County of North Ostrobothnia, 90220 Oulu, Finland; 4 Department of Obstetrics and Gynecology, Helsinki University Hospital, University of Helsinki, 00290 Helsinki, Finland

**Keywords:** PCOS, oral combined hormonal contraception, vaginal combined hormonal contraception, metabolic effects, OGTT

## Abstract

This clinical trial aims to compare hormonal and metabolic changes after a 9-week continuous use of oral or vaginal combined hormonal contraceptives (CHCs) in women with polycystic ovary syndrome (PCOS). We recruited 24 women with PCOS and randomized them to use either combined oral (COC, *n* = 13) or vaginal (CVC, *n* = 11) contraception. At baseline and 9 weeks, blood samples were collected and a 2 h glucose tolerance test (OGTT) was performed to evaluate hormonal and metabolic outcomes. After treatment, serum sex hormone binding globulin (SHBG) levels increased (*p* < 0.001 for both groups) and the free androgen index (FAI) decreased in both study groups (COC *p* < 0.001; CVC *p* = 0.007). OGTT glucose levels at 60 min (*p* = 0.011) and AUCglucose (*p* = 0.018) increased in the CVC group. Fasting insulin levels (*p* = 0.037) increased in the COC group, and insulin levels at 120 min increased in both groups (COC *p* = 0.004; CVC *p* = 0.042). There was a significant increase in triglyceride (*p* < 0.001) and hs-CRP (*p* = 0.032) levels in the CVC group. Both oral and vaginal CHCs decreased androgenicity and tended to promote insulin resistance in PCOS women. Larger and longer studies are needed to compare the metabolic effects of different administration routes of CHCs on women with PCOS.

## 1. Introduction

Polycystic ovary syndrome (PCOS) is the most common endocrine disorder, affecting 5–18% of women of reproductive age [1,2]. According to the Rotterdam criteria [3] and the international evidence-based PCOS guideline [4], PCOS is defined by at least two of the following three features: (1) polycystic ovaries on gynecological ultrasonography, (2) oligo- or anovulation, and/or (3) clinical and/or biochemical hyperandrogenism (hirsutism or high serum testosterone or androgen levels). Women with PCOS display an increased risk for glucose metabolism disorders [5,6], obesity, hypertension, dyslipidemia, insulin resistance (IR), and metabolic syndrome [5,6,7,8].

Combined hormonal contraceptives (CHCs) are the first-line treatment for the most common PCOS-related clinical manifestations, namely menstrual irregularity and hirsutism [4,9]. However, CHCs are known to induce unfavorable metabolic effects, especially on glucose metabolism, in the general population [10,11,12,13]. Recommendations for CHC use by women with PCOS are generally based on studies of women without PCOS, with a limited number of studies on the use of CHCs in PCOS. Given the metabolic burden related to PCOS, it is plausible that CHCs may worsen already existing metabolic disorders related to the syndrome [14]. Some studies of women without PCOS have indicated that the use of combined vaginal contraception (CVC) causes fewer metabolic effects than combined oral contraception (COC) [12,15], although the findings are inconsistent [13]. Given the widespread use of CVC among women and possibly less adverse systemic effects of vaginal administration due to lower hepatic stress compared with oral administration, there is a necessity for studies to evaluate the metabolic effects of CVC use in women with PCOS. 

Although the international evidence-based PCOS guideline recommends COCs in first-line pharmacological management for menstrual irregularity and hyperandrogenism, it does not provide specific recommendations for dosage, mode of administration, or specific preparation [4]. Earlier, the Amsterdam ESHRE/ASRM consensus on women’s health aspects of PCOS concluded that there is a need to perform head-to-head trials comparing different CHC strategies, as well as longitudinal follow-up studies on CHC use in women with PCOS [3].

The aim of this randomized controlled trial was to compare the hormonal and metabolic effects of COC and CVC in women with PCOS after nine weeks. 

## 2. Materials and Methods

This randomized, prospective, open-label, single-centered study was conducted at Oulu University Hospital, Finland, between 2011 and 2016. The study was approved by the Ethics Committee of Oulu University Hospital. All participants signed a written consent document. The study was registered at ClinicalTrials.gov (NCT01588873).

### 2.1. Study Population (Figure 1)

The participants were selected from the hospital register of Oulu University Hospital according to the ICD10 diagnosis code for PCOS (E 28.2). The women were eligible to participate if they were aged 18–40 years, healthy, and without medical contraindications for the use of CHCs (high blood pressure, migraine with focal aura, severe or multiple risk factors to thromboembolism, acute or chronic hepatocellular disease or hepatic adenomas or carcinomas, unexplained abnormal vaginal bleeding, diagnosed or suspected cancer, or an estrogen-dependent tumor), not using any medication, not smoking, not pregnant or breastfeeding, and had not used any hormonal or cortisone medicines for at least 2 months prior to study entry. 

**Figure 1:**
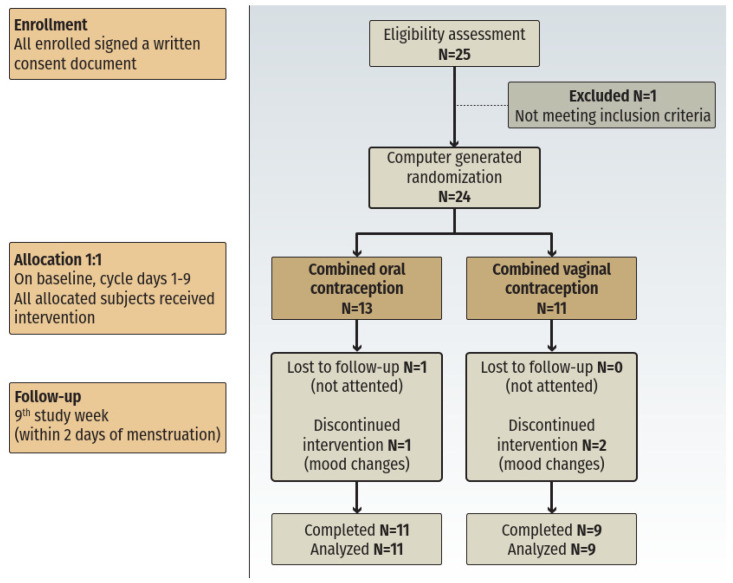
Flowchart of the study.

The participants were randomized to use either a combined hormonal oral contraceptive pill (COC group: ethinylestradiol, EE, 20 µg and desogestrel, 150 µg; Mercilon^®^; Organon Ltd., Dublin, Ireland) or a combined hormonal contraceptive vaginal ring (CVC group: EE, 15 µg/day and etonogestrel, an active metabolite of desogestrel, 120 µg/day; NuvaRing^®^; N.V.Organon, Oss, Netherlands) continuous for 9 weeks. The randomization list (allocation 1:1) was computer-generated. The participants went through two clinical examinations, the first at baseline and the second at 9 weeks of treatment, which included a gynecological examination, a transvaginal ultrasound (endometrium thickness, ovarian volumes, and the number of follicles), blood sampling for hormonal and metabolic parameters, and an oral glucose tolerance test (OGTT). At baseline, the clinical examination was performed between cycle days 1–3 and, at the ninth study week, within 3 days from the beginning of menstruation after discontinuation of the contraceptive preparation.

In all, 24 women with PCOS were recruited, 13 in the COC group and 11 in the CVC group (Figure 1). Diagnosis of PCOS was made according to the Rotterdam criteria. The baseline characteristics of the participants are described in Table 1.

### 2.2. Oral Glucose Tolerance Test

The 75 g 2 h oral OGTT was performed after 12 h of fasting at baseline and 9 weeks. Blood samples were taken at 0, 30, 60, and 120 min. Glucose and insulin areas under the curve (glucose AUC and insulin AUC), the homeostatic model assessment of insulin resistance (HOMA-IR), the homeostatic model assessment of β-cell function (HOMA-2β), and whole-body insulin sensitivity (i.e., Matsuda index) [16] were calculated based on OGTT results to evaluate glucose tolerance, IR, and insulin sensitivity. 

Although the hyperinsulinemic-euglycemic glucose clamp is the gold standard for evaluating insulin sensitivity, it is costly, time-consuming, invasive, and requires staff. The calculated indexes, such as HOMA-IR and Matsuda, have been shown to estimate insulin resistance and sensitivity more easily [16,17,18]. 

HOMA-IR (=insulin (mU/L) × glucose (mmol/L)/22.5) is a calculated index used to quantify IR from basal glucose and insulin levels and was first described in 1985 by Matthews et al. [18]. A strong linear correlation of HOMA-IR with the clamp has been found [17,18]. In women with PCOS, HOMA-IR has been used in various studies of different populations to assess IR [19,20,21,22] and has proven to be a robust clinical and epidemiological tool for assessing IR. HOMA-2β (=20 × fasting insulin (μIU/mL)/fasting glucose (mmol/mL) − 3.5) has been used as a marker of basal insulin secretion by pancreatic β-cells [21]. 

The Matsuda index (=[10,000/√fasting glucose × fasting insulin) (mean glucose (OGTT) × mean insulin OGTT)]) was described by Matsuda and DeFronzo in 1999. It estimates whole-body physiological insulin sensitivity [16]. In women with PCOS, the Matsuda index correlates well with HOMA-IR and the quantitative insulin-sensitivity check index (QUICKI), which indicates its reliability in the detection of IR [23,24]. 

### 2.3. Assays

Serum samples for the assay of total testosterone (T) were conducted by using Agilent triple quadrupole 6410 liquid chromatography–mass spectrometry (LC-MS) equipment with an electrospray ionization source operating in positive-ion mode (Agilent Technologies, Wilmington, DE, USA). Multiple reaction monitoring was used to quantify T by using trideuterated T (d3-T) with the following transitions: *m*/*z* 289.2 to 97 and 289.2 to 109 for T and 292.2 to 97 and 292.2 to 109 for d3-T. The intra-assay coefficients of variation (CVs) of the method were 5.3%, 1.6%, and 1.2% for T at 0.6, 6.6, and 27.7 nmol/L, respectively.

Sex hormone binding globulin (SHBG) was analyzed by chemiluminometric immunoassays (Immulite 2000, Siemens Healthcare Diagnostics, Los Angeles, CA, USA) with a sensitivity of 0.02 nmol/L. Serum glucose, total cholesterol, low-density lipoprotein cholesterol (LDL-C), high-density lipoprotein cholesterol (HDL-C), and triglycerides were assayed using an automatic chemical analyzer (Advia, 1800; Siemens Healthcare Diagnostics, Tarrytown, NY, USA), insulin by using an automated a chemiluminescence system (Advia Centaur; Siemens Healthcare Diagnostics, Tarrytown, NY, USA), and high-sensitivity C-reactive protein (hs-CRP) by using an immunonephelometry (BN ProSpec; Siemens Healthcare Diagnostics, Marburg, Germany). All samples (baseline and 9 weeks) from the same subject were analyzed in the same assay.

### 2.4. Statistical Analyses

Power calculation was based on our previous study comparing the metabolic effects of the same preparations (Mercilon^®^ and Nuvaring^®^) in young healthy women [13]. That study showed a significant increase of 0.44 mmol in serum triglyceride levels at 9 weeks of treatment with both preparations. The power analysis indicated that 17 women would have been needed in both study groups to reveal a similar increase in the serum level of triglycerides. To allow for dropouts, the planned sample size was 40 women (20 women in each group). Unfortunately, because of the strong criticism at the time of the recruitment raised in the media toward the thromboembolic risks linked to the use of hormonal contraception, the recruitment was extremely slow, and we managed eventually to recruit 24 women, 13 in the COC group and 11 in the CVC group. 

All variables are present as means with standard deviation (SD) in Table 2. Paired samples *t*-tests were performed for normally distributed variables and Wilcoxon’s tests were used for variables with a skewed distribution to explore changes in hormonal and metabolic levels within the same study group at the baseline and during the treatment. To analyze the differences between the study groups and the change from baseline to the 9th study week, we used a linear mixed model (repeated measures) with a random intercept. All results were adjusted with the BMI and age of the participants. 

Statistical analyses were performed using the Statistical Package for the Social Sciences (SPSS) software (version 28.0 for Windows, SPSS Inc., Chicago, IL, USA). The statistical significance level was set at *p* ≤ 0.05.

## 3. Results

### 3.1. Anthropometric Parameters 

At baseline, women in the COC group were older (32.4 vs. 30.8 years, *p* = 0.051), and their BMI tended to be higher (25.4 vs. 23.5, *p* = 0.068) compared to the CVC group (Table 1). At baseline and 9 weeks of treatment, there were no significant differences between the two study groups regarding BMI (*p* = 0.245), waist circumference (WC, *p* = 0.812), diastolic blood pressure (dBP, *p* = 0.550), or systolic blood pressure (sBP, *p* = 0.613) (Table 2).

### 3.2. Serum Levels of Androgens and SHBG 

There were no significant differences in serum levels of testosterone at 9 weeks between the groups. However, SHBG levels increased significantly in both groups (COC *p* < 0.001, CVC *p* < 0.001) between baseline and week 9, and FAI was decreased in both groups (COC *p* < 0.001, CVC *p* = 0.007) (Table 3). The changes in SHBG and FAI between baseline and week 9 were similar within the groups (Table 2).

### 3.3. Oral Glucose Tolerance Test (OGTT) 

**Figure 2:**
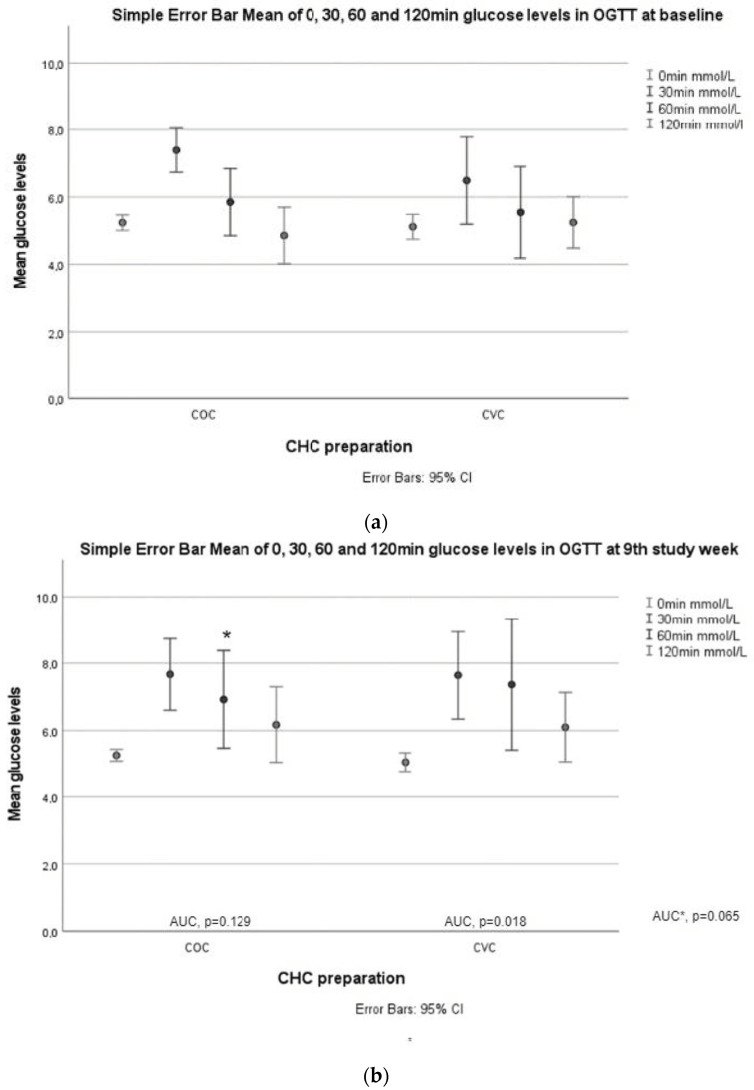

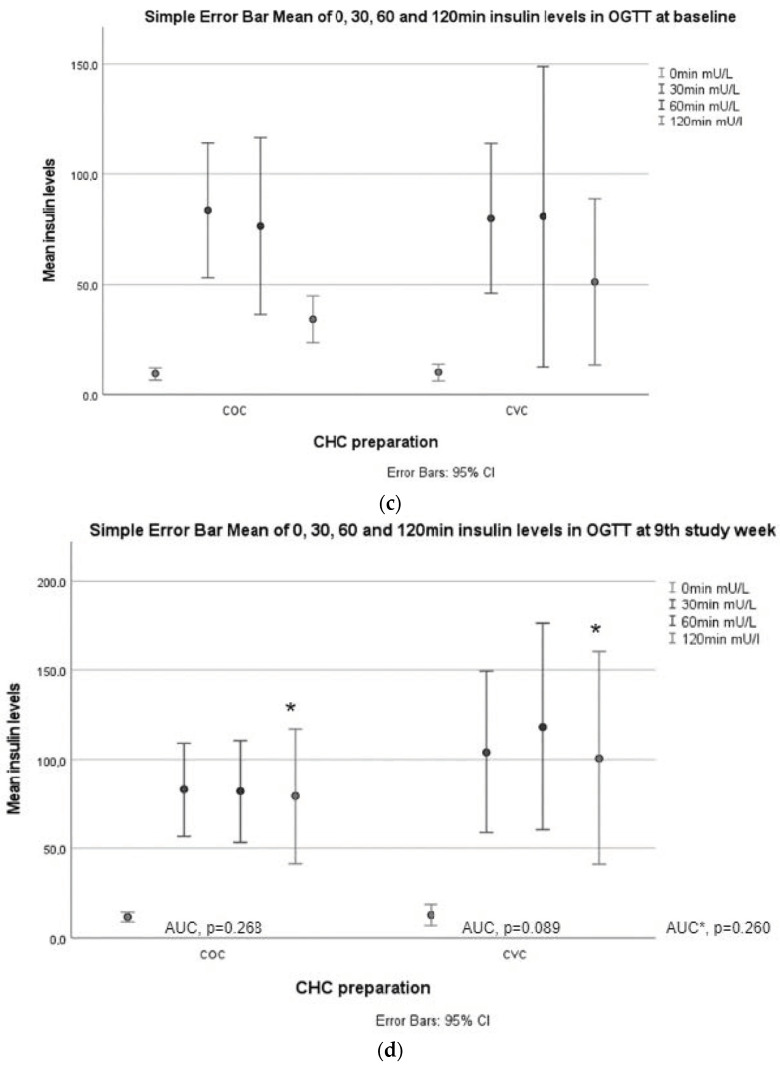
Glucose and insulin responses during OGTT. (**a**) Glucose values at 0, 30, 60, and 120 min in both study groups at baseline; (**b**) glucose values at 0, 30, 60, and 120 min in both study groups week 9. AUCglucose values between baseline and the ninth week in the COC and CVC groups. AUC * is AUCglucose between the study groups. An asterisk (*) marks a significant increase in 60 min glucose value in the CVC group compared to baseline; (**c**) insulin values at 0, 30, 60, and 120 min in both study groups at baseline.; (**d**) insulin values at 0, 30, 60, and 120 min in both study groups at baseline. AUCinsulin values between baseline and the ninth week in the COC and CVC groups. AUC * is AUCinsulin between the study groups at week 9. An asterisk (*) marks a significant increase in 120 min insulin values in both groups compared to baseline.

In the OGTT, glucose levels at 60 min were higher after 9 weeks of treatment compared to baseline (*p* = 0.008, adjusted *p* = 0.011) in the CVC group. Further, glucose AUC was increased significantly in the CVC group (*p* = 0.018, adjusted *p* = 0.034) (Table 3, Figure 2).

Fasting insulin levels (*p* = 0.037, adjusted *p* = 0.023) increased after 9 weeks of treatment in the COC group, and insulin levels at 120 min increased in both groups (COC *p* = 0.005, adjusted *p* = 0.004; CVC *p* = 0.028, adjusted *p* = 0.042) (Table 3, Figure 2).

HOMA-2β levels increased significantly in the COC group (*p* = 0.011), but the change became nonsignificant after adjustments (adjusted *p* = 0.10) (Table 3). HOMA-2β differed significantly between the two groups (*p* = 0.037, adjusted *p* = 0.030), but the increase in HOMA-2β levels was similar in both groups (Table 2). 

### 3.4. Serum Lipids and hs-CRP

Serum levels of triglycerides (*p* < 0.001, adjusted *p* < 0.001) increased significantly at 9 weeks of treatment in the CVC group (Table 3). There were differences in triglyceride levels (*p* = 0.030, adjusted *p* = 0.103) between baseline and 9 weeks; the levels increased, and the change differed between the groups (*p* = 0.542, adjusted *p* = 0.046) (Table 2). 

Hs-CRP levels (*p* = 0.032, adjusted *p* = 0.040) increased significantly after 9 weeks in the CVC group (Table 3). The levels differed between the groups after adjustments (adjusted *p* = 0.021), but the change in hs-CRP was similar in both groups (*p* = 0.376, adjusted 0.382) (Table 2). 

## 4. Discussion

In this study, both COC and CVC decreased androgenicity but displayed only mild effects on glucose metabolism, IR, lipid profile, and chronic inflammation. Though the study failed to recruit the targeted number of women, the data contrasted with our hypothesis, as CVC did not seem to have a more beneficial hormonal or metabolic profile than COC in PCOS.

CHC preparations are the most used medical treatment for PCOS, as they reduce menstrual abnormalities and relieve manifestations of clinical hyperandrogenism (acne and hirsutism). In the present study, a 9-week use of 20 μg EE + DSG or 15 μg/d EE/EGS caused an approximately 200–270% increase in SHBG levels and a subsequent 74–84% decrease in FAI levels, in line with the results of previous studies [13,15,25]. Some studies have reported a greater increase in SHBG during oral CHC use [13,26], whereas others have found that SHBG increased more during CVC treatment [15,25]. The present results suggest that the routes are comparably efficient in improving hyperandrogenemia in PCOS.

In some studies, CVC was considered to cause fewer adverse changes in glucose metabolism and insulin sensitivity in general female populations [15]. In the present study, after 9 weeks of treatment, AUCglucose increased in the CVC group. A small compensatory increase in insulin secretion was observed in both groups. In the COC group, fasting insulin and 120 min insulin levels increased and, in the CVC group, there was an increase in 120 min insulin levels. These results are partly in line with those of our previous study, which demonstrated a reduction in insulin sensitivity and an increase in AUCglucose levels among 54 healthy young women who were given oral, vaginal, or transdermal CHCs continuously for 9 weeks [13]. However, not all studies agree on the detrimental effect of CHCs on glucose metabolism. In one study, CVC users did not experience any changes in carbohydrate metabolism compared to COC users over five cycles [15]. Furthermore, CVC for 24 months was found to be safe in women with type 1 diabetes when estimated by glycosylated hemoglobin levels [27]. Moreover, in the only randomized study performed with women with PCOS (*n* = 37), CVC improved insulin sensitivity and glucose tolerance, whereas COC (containing EE + drospirenone) worsened IR and insulin secretion at 6 months of treatment [28]. Comparisons with previous studies are challenging because studies differ regarding CHC preparations and the methods used to evaluate glucose metabolism and IR. The gold standard for assessing IR is the hyperinsulinemic-euglycemic glucose clamp technique. However, several measurements based on serum glucose and insulin response to glucose intake, such as HOMA-IR, HOMA-2β, and the Matsuda index, have been shown to be useful. These indexes have been shown to reliably reflect IR in several previous studies in women with PCOS [19,20,21,22]. On the other hand, it is unclear whether patients presenting mild IR (i.e., an altered response to a clamp but a normal HOMA-IR and a normal response to an oral glucose test) display any additional clinical problems when compared to patients with a normal response to the clamp. Indeed, there are also concerns that the evaluation of IR in women with PCOS is method-dependent, and there may be discrepancies between markers [29]. In addition, lean women with PCOS may be more easily misclassified as insulin sensitive [30]. Nonetheless, our results raise concerns that CVC may not be safer than COC in women with PCOS regarding its effects on glucose metabolism. 

In the present study, changes in lipid profile showed an increase in triglyceride levels in the CVC group, and the increase was slightly greater in the CVC group. These results align with those of previous studies among women with PCOS, showing that COC use is associated with increased levels of triglycerides but also HDL and total cholesterol levels [14,31]. Similar results were generated in a study performed on healthy women treated with oral, transdermal, or vaginal CHCs for 9 weeks, showing an increase in triglycerides and HDL in all study groups [13]. Comparably, oral EE administration has been shown to result in increased total cholesterol, mainly due to increased HDL cholesterol and triglycerides [32,33,34]. A similar effect during the use of CVC has also been described [35]. High triglyceride levels seem to be an independent predictor of the future risk of myocardial infarction [36] and have been associated with elevated cardiovascular risks [37], specifically in women [37]. A meta-analysis of CHC effects on the lipid profile in PCOS women concluded that desogestrel containing CHCs increased triglyceride levels after 6 months of treatment [33]. In the present study, triglyceride levels e increased significantly in the CVC group. A 9-week study is too short for solid conclusions regarding lipid profile changes with COC or CVC use. However, our results do not support the recommendation that vaginal CHC be preferred to oral preparations to decrease the cardiovascular risks linked to PCOS. As an abnormal lipid profile is typically present in women with PCOS [38], larger, long-lasting follow-up studies and real-world register data analyses are needed to clarify whether the use of CHCs (either oral or vaginal) will increase the risk of cardiovascular morbidity and mortality in women with PCOS. It is possible that the alleviation of hyperandrogenemia with CHCs overcomes mild impairments in metabolic parameters.

Serum levels of hs-CRP increased in the CVC group after 9 weeks of treatment. Despite differences in CRP levels, the change in hs-CRP levels was similar between the two groups. This result is in line with studies performed with oral and vaginal preparations [39,40] and with our recently published data showing EE to be a strong promoter of chronic low-grade inflammation [13,41]. This is an important finding, as women with PCOS have been shown to display chronic inflammation [42,43], which, in turn, is associated with an increased risk of cardiovascular diseases and events, as well as overall mortality [44]. Of note, the findings of a recent study comparing estradiol valerate (EV) with EE among healthy women suggest that EV could display a more neutral effect on inflammation and lipids [41]. Further randomized studies are needed to clarify whether preparations containing EV instead of EE could be safer regarding cardiovascular risks in women with PCOS.

### Strengths and Limitations

The main strengths of the present study are that PCOS diagnosis was made by a gynecologist and the participants were homogeneous concerning ethnicity, as all were Caucasians. All in all, the study provides important data for future meta-analyses. The weaknesses of the study are the failure to recruit enough participants to meet the power calculation criteria for a sufficient sample size and to engage participants to finish the study, which underscores the challenge of running a randomized clinical trial. Additionally, a slightly higher BMI (nonsignificant) and age at baseline in the COC group may have influenced the results. Further, the power calculation was based on changes in triglycerides, not on glucose metabolism parameters or inflammation markers, which must be taken into consideration when interpreting the results. Additionally, as discussed earlier, the hyperinsulinemic-euglycemic glucose clamp technique is the gold standard for IR measurement, but it is costly, time-consuming, invasive, and requires staff. We used basal and OGTT-derived calculated indexes (HOMA-IR, HOMA-2β, and the Matsuda index) to evaluate IR in women with PCOS. Lastly, the short follow-up period does not permit conclusions to be drawn on the long-term consequences of the use of COC or CVC, warranting future studies. 

## 5. Conclusions

Contrary to our hypothesis, CVC did not seem to be metabolically safer than COC based on this short clinical study. As there are a limited number of studies assessing different administration routes of CHCs for women with PCOS, the results show some new data and underline the need for larger and longer studies comparing the metabolic effects of CHC administration routes in PCOS. 

## Figures and Tables

**Table 1:** Baseline characteristics of the study population.

	COC	CVC	*p*-Value *
Age (years)	32.4 ± 6.6	30.8 ± 4.9	0.051
BMI (kg/m^2^)	25.4 ± 3.3	23.2 ± 2.4	0.068
PCOM	13/13	11/11	0.287
oligomenorrhea	10/13	9/11	0.493
hirsutism/high testo	6/13	5/11	0.974

* *p*-value between the study groups at baseline. COC, combined oral contraceptive; CVC, combined vaginal contraceptive; BMI, body mass index; PCOM, polycystic ovarian morphology.

**Table 2:** Differences in the parameters of androgen secretion, glucose metabolism, lipid profile, and inflammation between the study groups. Analyses were performed with a linear mixed model with a random intercept.

Variable	Fixed Effect	Estimate	95%CI	*p*-Value	*p* Adjusted *
BMI (kg/m^2^)	time	0.33	−0.08; 0.75	0.128	0.109
	CHC	−1.70	−4.20; 0.79	0.131	0.170
	time*CHC	−0.34	−0.95; 0.26	0.245	0.244
WC (cm)	time	−0.36	−3.32; 2.61	0.748	0.825
	CHC	−0.81	−8.22; 6.60	0.626	0.825
	time*CHC	0.42	−3.99; 4.83	0.812	0.843
sBP (mmHg)	time	−0.32	−5.77; 5.14	0.905	0.394
	CHC	−7.07	−16.41; 2.28	0.134	0.195
	time*CHC	−2.42	−10.76; 5.92	0.550	0.880
dBP (mmHg)	time	−0.63	−4.99; 3.73	0.425	0.762
	CHC	−2.78	−11.02; 5.45	0.109	0.495
	time*CHC	−1.53	−7.94; 4.88	0.613	0.618
SHBG	time	−111.8	−144.98; −78.7	<0.001	<0.001
	CHC	37.8	−6.6; 81.9	0.019	0.093
	time*CHC	−39.2	−88.5; 10.1	0.080	0.113
Kol (mmol/L)	time	−111.8	−144.98; −78.7	<0.001	<0.001
	CHC	37.8	−6.6; 81.9	0.019	0.093
	time*CHC	−39.2	−88.5; 10.1	0.080	0.113
LDL-C (mmol/L)	time	−0.188	−0.61; 0.23	0.569	0.360
	CHC	0.113	−0.52; 0.75	0.766	0.718
	time*CHC	0.149	−0.48; 0.78	0.598	0.622
HDL-C (mmol/L)	time	−0.025	−0.34; 0.29	0.076	0.869
	CHC	0.291	−0.34; 0.92	0.574	0.351
	time*CHC	0.052	−0.42; 0.53	0.595	0.821
triglycerides (mmol/L)	time	−0.179	−0.4; 0.04	0.032	0.103
	CHC	0.236	−0.12; 0.59	0.919	0.182
	time*CHC	−0.333	−0.66; −0.01	0.542	0.046
CRP (mmol/L)	time	−0.538	−1.96; 0.88	0.403	0.432
	CHC	2.139	0.35; 3.93	0.109	0.021
	time*CHC	−1.350	−3.44; 0.74	0.376	0.190
fasting glucose (mmol/L)	time	−0.099	−0.36–0.17	0.700	0.440
	CHC	−0.181	−0.50–0.14	0.213	0.266
	time*CHC	0.131	−0.26–0.52	0.456	0.488
fasting insulin (mU/L)	time	−2.215	−4.8; 0.39	0.140	0.091
	CHC	3.127	−4.8; 0.39	0.718	0.108
	time*CHC	0.749	−3.13; 4.63	0.899	0.690
AUCglucose	time	−1.666	−3.52; 0.19	0.107	0.817
	CHC	0.283	−2.19; 2.75	0.065	0.076
	time*CHC	0.413	−2.42; 3.25	0.921	0.764
AUCinsulin	time	−27.11	−70.7; 16.5	0.260	0.206
	CHC	71.37	−1.8; 144.5	0.363	0.056
	time*CHC	−20.00	−86.7; 46.7	0.249	0.534
HOMA-IR	time	−0.430	−1.20; 0.34	0.404	0.266
	CHC	1.178	0.18; 2.18	0.295	0.022
	time*CHC	−0.292	−1.5;0.91	0.316	0.617
HOMA-2β	time	−26.61	−55.3; 2.05	0.269	0.067
	CHC	49.15	5.1; 93.2	0.065	0.030
	time*CHC	2.428	−40.2; 45.1	0.827	0.906
Matsuda index	time	1.835	0.29; 3.38	0.065	0.023
	CHC	0.302	−2.95; 3.55	0.673	0.850
	time*CHC	−0.968	−3.29; 1.36	0.717	0.759

* Adjusted with BMI and age. Time comparison between baseline and the ninth week of study. CHC, comparison between study groups. Time*CHC, comparison between study groups between baseline and week 9. COC, combined oral contraceptive; CVC, combined vaginal contraceptive; BMI, body mass index; WC, waist circumference; sBP, systolic blood pressure; dBP, diastolic blood pressure; SHBG, sex hormone binding globulin; FAI, free androgen index; HOMA-IR, homeostasis model assessment of insulin resistance; HOMA-2β, homeostasis model assessment of β-cell function; HDL-C, high-density lipoprotein cholesterol; LDL-C, low-density lipoprotein cholesterol; hs-CRP, high-sensitivity C-reactive protein.

**Table 3:** Parameters (mean ± standard deviation, SD) related to androgen secretion, glucose metabolism, lipid profile, and inflammation in the study groups.

	COC					CVC				
	Week 0	Week 9	Change	Pcoc	Padj	Week 0	Week 9	Change	Pcvc	Padj
*n*	13	11				11	9			
BMI (kg/m^2^)	25.4 ± 3.3	25.1 ± 3.0	−0.3 (−0.6; 0.1)	0.173	0.157	23.2 ± 2.4	23.5 ± 2.7	0.02 (−0.3; 0.4)	0.917	0.929
WC (cm)	82.3 ± 10.9	82.1 ± 8.6	−0.4 (−3.3; 2.5)	0.842	0.812	80.9 ± 7.5	80.5 ± 8.1	0.00 (−1.1; 1.1)	0.990	0.912
sBP (mmHg)	116.3 ± 9.4	116.4 ± 13.3	0.5 (−6.6; 7.5)	0.928	0.876	106.8 ± 10.1	111.1 ± 7.7	1.9 (−2.7; 6.4)	0.270	0.322
dBP (mmHg)	69.4 ± 8.2	70.5 ± 8.6	1.6 (−2.9; 6.1)	0.472	0.656	62.0 ± 9.2	65.9 ± 7.8	2.5 (−1.9; 6.9)	0.167	0.203
Testo (nmol/L)	1.31 ± 0.5	1.13 ± 0.6	−0.1 (−0.5; 0.2)	0.331	0.450	2.31 ± 2.1	1.20 ± 0.3	−1.3 (−3.2; 0.6)	0.140	0.195
SHBG (nmol/L)	48.54 ± 17.5	156.36 ± 1.6	109.8 (84.1; 135.6)	<0.001	<0.001	55.73 ± 22.3	208.84 ± 84.0	151.6 (101.1; 202.1)	<0.001	<0.001
FAI	2.88 ± 1.3	0.75 ± 0.4	−2.1 (−3.3; −1.0)	<0.001	<0.001	3.80 ± 2.9	0.61 ± 0.2	−3.5 (−6.0; −1.0)	0.005	0.007
Fasting glucose (mmol/L)	5.3 ± 0.3	5.3 ± 0.3	0.0 (−0.3; 0.3)	0.938	0.863	5.2 ± 0.5	5.1 ± 0.4	−0.2 (−0.4; 0.1)	0.166	0.324
Fasting insulin (mU/L)	10.1 ± 5.1	11.7 ± 4.1	1.58 (0.02; 3.1)	0.037	0.020	11.1 ± 6.2	12.8 ± 7.7	1.72 (−2.1; 5.6)	0.255	0.168
AUCglucose	12.2 ± 7.1	13.1 ± 10.6	1.5 (−1.4; 4.3)	0.129	0.281	10.7 ± 8.8	13.7 ± 11.5	1.5 (0.3; 2.7)	0.018	0.034
AUCinsulin	92.0 ± 286.6	129.0 ± 199.2	14.4 (−18.9; 47.6)	0.268	0.240	72.3 ± 402.0	212.7 ± 339.7	44.0 (−18.9; 106.8)	0.089	0.294
HOMA-IR	2.3 ± 1.3	2.7 ± 0.9	0.25 (−0.2; 0.7)	0.189	0.235	2.3 ± 1.5	3.4 ± 1.9	0.87 (−0.5; 2.3)	0.134	0.212
HOMA-2β	106.1 ± 41.7	137.0 ± 53.8	26.1 (6.2; 46.1)	0.011	0.010	130.0 ± 60.8	163.4 ± 81.5	30.7 (−9.4; 70.7)	0.098	0.532
Matsuda index	6.09 ± 3.4	4.91 ± 1.6	−1.2 (−2.4; 0.1)	0.066	0.089	6.18 ± 3.1	5.39 ± 3.6	−0.8 (−2.8; 1.1)	0.241	0.288
Cholesterol (mmol/L)	4.15 ± 0.6	4.33 ± 0.7	0.04 (−0.5; 0.6)	0.572	0.722	4.29 ± 0.5	4.36 ± 0.7	0.14 (−1.1; 1.4)	0.749	0.787
HDL-C (mmol/L)	1.44 ± 0.4	1.67 ± 0.5	−0.25 (−0.9; 0.4)	0.088	0.113	1.65 ± 0.3	1.65 ± 0.3	0.03 (−0.2; 0.3)	0.869	0.985
LDL-C (mmol/L)	2.56 ± 0.7	2.33 ± 0.8	0.19 (−0.04; 0.4)	0.959	0.988	2.60 ± 0.6	2.64 ± 0.7	−0.02 (−0.2; 0.3)	0.943	0.098
Triglycerides (mmol/L)	0.93 ± 0.4	1.29 ± 0.8	0.36 (−0.1; 0.8)	0.098	0.367	0.76 ± 0.3	1.28 ± 0.5	0.49 (0.3; 0.7)	<0.001	<0.001
Hs-CRP (mmol/L)	1.58 ± 1.8	2.00 ± 2.1	0.78 (−0.46; 2.0)	0.302	0.567	1.65 ± 1.5	3.50 ± 2.3	1.75 (−0.02; 3.6)	0.032	0.040

COC, combined oral contraceptive; CVC, combined vaginal contraceptive; BMI, body mass index; WC, waist circumference; sBP, systolic blood pressure; dBP, diastolic blood pressure; SHBG, sex hormone binding globulin; FAI, free androgen index; HOMA-IR, homeostasis model assessment of insulin resistance; HOMA-2β, homeostasis model assessment of β-cell function; HDL-C, high-density lipoprotein cholesterol; LDL-C, low-density lipoprotein cholesterol; hs-CRP, high-sensitivity C-reactive protein.

## Data Availability

Data cannot be shared openly but are available on request from authors upon reasonable request.
